# Association of cytochromes P450 3A4*22 and 3A5*3 genotypes and polymorphism with response to simvastatin in hypercholesterolemia patients

**DOI:** 10.1371/journal.pone.0260824

**Published:** 2022-07-15

**Authors:** Elbatool G. Elalem, Musharraf Jelani, Alaa Khedr, Aftab Ahmad, Tareef Y. Alaama, Mohamed Nabeel Alaama, Huda M. Al-Kreathy, Zoheir A. Damanhouri

**Affiliations:** 1 Department of Pharmacology, Faculty of Medicine, King Abdulaziz University, Jeddah, Saudi Arabia; 2 Department of Genetic Medicine, Faculty of Medicine, King Abdulaziz University, Jeddah, Saudi Arabia; 3 Princess Al-Jawhara Center of Excellence in Research of Hereditary Disorders, King Abdulaziz University, Jeddah, Saudi Arabia; 4 Department of Analytical Chemistry, Faculty of Pharmacy, King Abdulaziz University, Jeddah, Saudi Arabia; 5 Health Information Technology Department, Jeddah Community College, King Abdulaziz University, Jeddah, Saudi Arabia; 6 Department of Medicine, Faculty of Medicine, King Abdulaziz University, Jeddah, Saudi Arabia; King Saud University, SAUDI ARABIA

## Abstract

**Backgrounds:**

Inter-individual variability in response to statin was mainly due to genetic differences. This study aimed to investigate the association of CYP3A4*22 (rs35599367), CYP3A5*3 (rs776746) single nucleotide polymorphism (SNP) with response to simvastatin in hypercholesterolemia patients conducted at King Abdulaziz University hospital (KAUH) in Jeddah, Saudi Arabia.

**Patients and methods:**

A total of 274 participants were registered in the current study. Hypercholesterolemic patients taking simvastatin 20 mg (n = 148) and control subjects (n = 126) were tested for rs35599367 and rs776746 genotypes using Custom Taqman ® Assay Probes. Response to simvastatin in these patients was assessed by determination of low density lipoprotein (LDL-C), total cholesterol (TC) and by measuring statin plasma levels using Liquid Chromatography-Mass Spectrometry (LC-MS).

**Results:**

None of the participants carried a homozygous CYP3A4*22 mutant genotype, while 12 (4.4%) individuals had a heterozygous genotype and 262 (95.6%) had a wild homozygous genotype. The CYP3A5*3 allele was detected in the homozygous mutant form in 16 (5.8%) individuals, while 74 (27.0%) individuals carried the heterozygous genotype and 184 (67.2%) carried the wildtype homozygous genotype. Of the patient group, 15 (11%) were classified as intermediate metabolizers (IMs) and 133 (89%) as extensive metabolizers (EMs). Plasma simvastatin concentrations for the combined *CYP3A4/5* genotypes were significantly (P<0.05) higher in the IMs group than in the EMs group. TC and plasma LDL-C levels were also significantly (P<0.05) higher in IMs than in EMs.

**Conclusion:**

The present study showed associations between CYP3A4*22 (rs35599367) and CYP3A5*3 (rs776746) SNP combination genotypes with response to statins in hypercholesterolemia. Patients who had either a mutant homozygous allele for CYP3A5*3 or mutant homozygous and heterozygous alleles for CYP3A4*22 showed increased response to lower TC and LDL-C levels.

## Introduction

Hypercholesterolemia and its clinical manifestations such as cardiovascular diseases (CVDs) are the leading cause of death worldwide, both in developed industrialized and developing countries [1, 2]. According to the American Heart Association, it has been reported that by 2030, 43.9% of the United States population will have some form of CVD [3] In Greece, the CVDs accounted for 45.8% of all deaths in both men and women [4]. More than 90 genes affected lipid levels have been identified [5, 6]. Apart from genetic factors, some important risk factors are associated with the increased CVD risk, including non-modifiable risk factors such as gender, age and family history, while modifiable risk factors include increased plasma lipid concentration [7, 8], presence of type 2 diabetes mellitus (T2DM) [9, 3], hypertension [10, 11], smoking [12], alcohol consumption and some environmental and lifestyle factors [13, 14]. One of the independent risk factors for CVD is dyslipidemia, therefore, lowering low-density lipoprotein cholesterol (LDL-C) and total cholesterol (TC) along with reducing or/and modifying other modifiable risk factors is a primary goal in the optimal treatment and prevention of CVD [15, 16].

The main group of drugs used as standard and first-line therapy in patients with hypercholesterolemia are statins, also known as 3-hydroxy,3-methylglutaryl coenzyme A (HMG-CoA) reductase inhibitors [17, 18]. According to the various clinical trials, the percentage risk reduction of cardiovascular disease by statins is 20–50%, which is strongly associated with the reduction of LDL-C [19, 20]. Despite the correct dose titration of statins and their clinical efficacy to achieve the desired response, persistently elevated LDL-C levels above the recommended target remain in approximately one-third of patients, leading to interindividual variability in response to statins [21, 22]. This interindividual variability due to the genetic factor accounts for nearly 20–95%, suggesting the implementation of genotype and phenotype testing to predict and improve drug response [23–27].

Among the available statins in the market, simvastatin, atorvastatin, and lovastatin are metabolized by cytochrome *P450 3A5* (*CYP3A5*) and cytochrome *P450 3A4* (*CYP3A4*), [28]. The extent to which either of these enzymes (*CYP3A4* and *CYP3A5*) is responsible for statin metabolism depends on the dose of statin administered and the patient [29]. Since it has been reported that there is a strong association between the administered statin dose, statin blood level and the resulting lipid response, any genetic polymorphism within *CYP3A4* is thought to affect the blood level of the drug, the kinetic disposition of statin metabolites and subsequently the lipid response [30, 31]. Several studies on different *CYP3A4* polymorphisms have reported interindividual variations in statin response associated with genetic differences, and occurs when a variant allele replaces one or both wild-type alleles [32, 33]. Variant alleles usually encode a *CYP450* enzyme that has reduced or no activity. Persons with two copies of variant alleles are poor metabolizers, those with one wild-type and one variant allele have reduced activity, whereas those who inherit multiple copies of wild-type alleles have excessive enzyme activity.

In Saudi Arabia, studies showed high rates of hypercholesterolemia in both Saudis and Non-Saudis residents. As a result, statins were highly prescribed to lower LDL-C levels. From the latest annual statistics book of the Ministry of Health in Saudi Arabia (MOH) for 2018 G, the number of emergency admissions due to heart disease in Saudi Arabia was 149,090 cases, with Saudi patients accounting for 122,477 cases. The number of deaths due to circulatory diseases was reported to be 8,924 cases in 2012 G [34]. Al-Nozha et al. conducted a study on 16,819 Saudi subjects between 1995 and 2000, which found that the prevalence of hypercholesterolemia was 54% [35]. More recent studies in 2018, Medani et al. in a cross-sectional study indicated the prevalence of hypercholesterolemia in a Saudi cohort was 45.3%, which increased with age reaching a maximum at the fifth decade [36]. From these studies, there is a high incidence of heart disease and hypercholesterolemia in Saudi society. This study investigated the influence of CYP3A4*22 (rs35599367), CYP3A5*3 (rs776746) SNPs and combined *CYP3A4/5* genotypes on the response to statins in hypercholesterolemic patients treated at King Abdulaziz University Hospital (KAUH), Jeddah, Saudi Arabia.

## Patients and methods

### Patients

Two hundred and seventy-four subjects were enrolled in this study and divided into two groups, either control subjects (126) (non-statin users) or hypercholesterolemic patients (148) taking simvastatin (Zocor® 20mg) and followed up at KAUH. All participants were of Arab ethnicity (Saudi and non-Saudi) having similar type of diet, life style and habit. Patients were excluded from lipid profile testing for genotyping: if they were taking other lipid-lowering medications such as fibrates, bile acid sequestrants, nicotinic acids, or ezetimibe; or if they were not taking the same simvastatin dose for at least six weeks prior to blood collection; or if they were taking medications that interact with simvastatin. Participants were genotyped for CYP3A4*22 and CYP3A5*3, and grouped into phenotypes because simvastatin is metabolized by both *CYP3A4* and *CYP3A5* due to high amino acid sequence similarity. With comparable expression of both CYPs in some individuals, the interaction between the two enzyme polymorphisms should be considered [37]. Depending on the presence of CYP3A4*22 and CYP3A5*3 alleles, patients in the current study were classified into three categories: poor metabolizers (PMs), intermediate metabolizers (IMs), and extensive metabolizers (EMs) [38, 39].

The classification of alleles expresser status for each CYP was done according to the following: for CYP3A4*22, (GG) allele genotype was considered as expresser allele, while, (AA) and (GA) allele genotypes were considered as a decrease of function (DOF) alleles. For CYP3A5*3, (CC) and (CT) allele genotypes were considered as expresser alleles, while, (TT) allele genotype was considered as the loss of function (LOF) allele (i.e., non-expresser). PMs are individuals who carry the reduced expresser allele for CYP3A4*22 and non-expresser allele for CYP3A5*3. They are the individuals who carry a DOF for CYP3A4*22 (either AA or GA) and LOF for CYP3A5*3 which is the (TT) allele. IMs are individuals who carry the expresser allele of either CYPs but not both. They are individuals who carry expresser allele of CYP3A4*22 (GG) and non-expresser allele of CYP3A5*3 (TT) allele, or those who carry DOF allele for CYP3A4*22 (either AA or GA) and the expresser alleles for CYP3A5*3 (either CC or CT). EMs are individuals who carry the expresser alleles of both CYPs. They are individuals who carried expresser allele of CYP3A4*22 (GG) and CYP3A5*3 (either CC or CT).

The protocol of the present study was approved by KAUH, Biomedical Ethics Research Committee. Written informed consents were obtained from all participants.

### Sample collection

Five milliliters (ml) of venous blood samples were obtained from each patient (2–4 hours after dosing with simvastatin) into tubes containing ethylene diamine tetraacetic acid (EDTA). The blood was immediately centrifuged for 15 minutes at 4,000 g and the collected plasma was preserved at -80°C until used for genotype and phenotype study.

### Molecular biology techniques

#### Genomic DNA isolation

DNA was extracted from blood samples leukocytes by DNA mini kit QIAamp (Qiagen- Alameda- CA, USA).

#### DNA quantification

DNA was quantified by spectrophotometer Nanodrop-2000 (Thermo Scientific, USA).

#### Taqman assay genotyping

Single nucleotide polymorphism (CYP3A4*22 rs35599367 C>T) and (CYP3A5*3 rs776746 G>A) genotypes were detected by Custom Taqman Assay Probes using Real time PCR system 7500 fast (Applied Biosystems, CA, USA). TaqMan Drug Metabolism Genotyping Assay (40X) was obtained from (Applied Biosystems, CA, USA). PCR Pre- and post-PCR fluorescence measurements and genotype calls were made using 7500 fast Real time PCR system.

#### 7500 Fast real time PCR condition

DNA was used from each patient in each 25 μl reaction (10–20 ng) according to standard PCR thermocycling conditions. This consisted of an initial cycle of 10 minutes at 95° C, followed by 40 cycles of denaturation at 92° C for 15 seconds, annealing and extension at 60° C for 1 minute.

### Determination of plasma statin concentration

**Fig 1:**
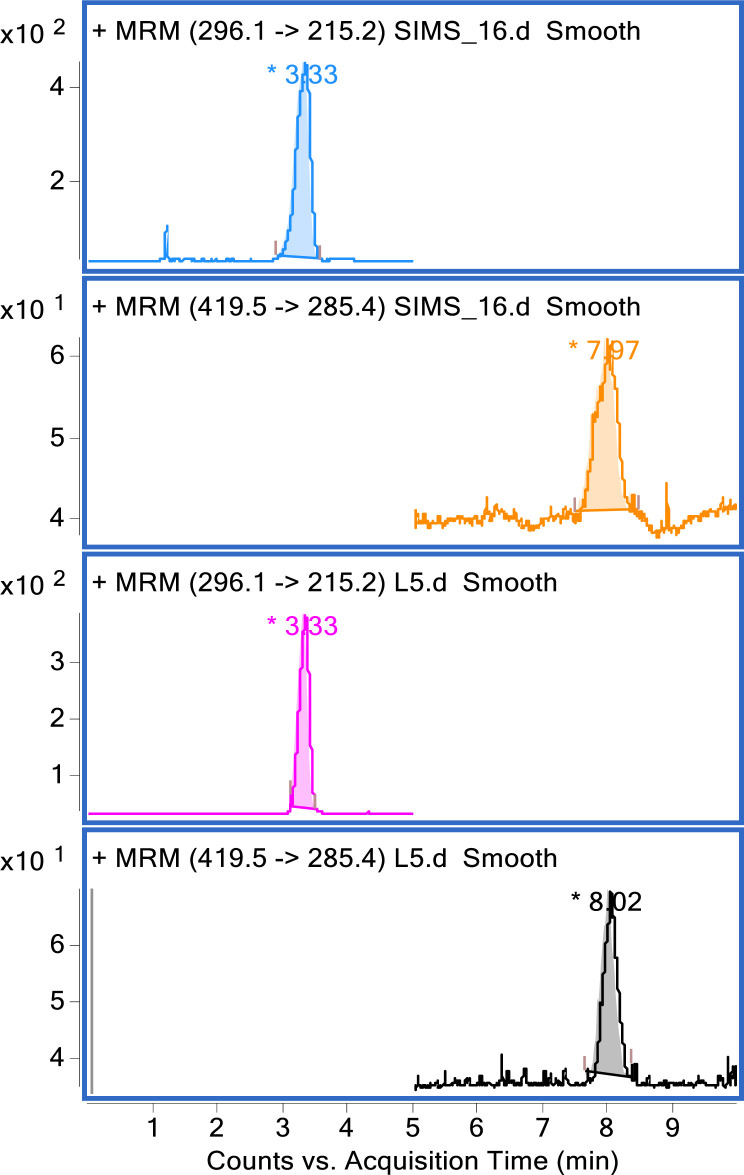
MRM chromatograms for simvastatin standard solution. (50 ng/ml) showing Simvastatin peak at 7.9 min (MRM, 419.5➔285.4) and In St peak at 3.3 min (MRM, 296.1➔215.2).

The high-performance liquid chromatography (HPLC) system comprised of Agilent 1200 system, autosampler, quaternary pump, solvent delivery mode, and column compartment (Agilent Technologies, Germany). Detector utilized an Agilent 6420 triple quad mass spectrometer (TQ-MS) which was coordinated by MassHunter software. The screw-capped (PTFE/silicon) total recovery 1-ml autosampler vial, 12 millimeter (mm) was used. The injection volume was 20 μL [40]. Chromatograms for simvastatin are shown in Fig 1.

### Determination of plasma TC and LDL-C concentrations

The levels of TC and LDL-C were determined by standard kit methods using fully Automated COBAS ® 8000 modular analyzer series at KAUH, Jeddah, Saudi Arabia.

### Statistical analysis

Data analysis was performed using the software Statistical Package for the Social Sciences (SPSS) version 20 (SPSS Inc., IL, USA). Results were expressed as mean +/- standard error or number (%). Differences in the mean of the variables studied, including clinical and demographic characteristics, were tested using an unpaired Student "t" test, analysis of variance (ANOVA), or chi-square (χ2) test, depending on the type and number of variables studied. A P value less than 0.05 was considered significant.

## Results

### Patients’ demographic data

The demographic data of the patients are summarized in Table 1. In the current study, a total of 274 participants were registered. Statin users were 148 (54%) and control non-statin users were 126 (46.00%). Male participants were 139 (51.00%) and females were 135 (49.00%). Saudis were 189 (69%) while 85 were non-Saudis (31%). All non-Saudi participants were of Arab ethnicity. The simvastatin dose used by all participants was 20mg/day.

**Table 1:** Demographic data for all participants.

	Simvastatin Users (20 mg)	Non-Statin Users (Control Group)	Total No.
Participants’ number	148 (54%)	126 (46%)	274 (100%)
Gender			
Male	75 (51%)	64 (51%)	139 (51.00%)
Female	73 (49%)	62 (49%)	135 (49.00%)
Age (years)	59.30 ± 8.24	52.36 ± 11.71	55.17 ± 12.71
Nationality			
Saudi	116 (78%)	73 (42%)	189 (69%)
Non-Saudi	32 (22%)	53 (42%)	85 (31%)

Data were expressed as mean +/- SEM or number (%) as appropriate.

### Genotypes frequencies and MAFs for CYP3A4*22 and CYP3A5*3 SNPs

TaqMan assay genotyping data for all individuals including mutant and wild-type genotypes for rs776746 (CYP3A5*3) alleles and for rs35599367 (CYP3A4*22) are summarized in Tables 2 and 3. Regarding the CYP3A4*22, the G allele was the most frequently observed in both Saudi (97.8%) and non-Saudi (97.6%) participants. Of all participants, 262 carried CYP3A4*22 wild homozygous (GG) with a genotype frequency of 95.60% and 12 participants carried heterozygous (GA) with a genotype frequency of 4.40%, with MAF 0.022. For the CYP3A5*3 SNP, C allele was the most frequent among both Saudi (77.0%) and non-Saudi (89.0%) participants. 184 participants carried wild homozygous (CC) with a genotype frequency of 67.20%; 16 participants carried mutant homozygous (TT) with a genotype frequency of 5.80% and 74 participants carried heterozygous (CT) with a genotype frequency of 27.00%, with MAF 0.193. The loci for rs35599367 (CYP3A4*22) was consistent with HWE (P = 0.701), indicating that the mutant allele was normally distributed in the population studied. While the loci for rs776746 (CYP3A5*3) was not consistent with HWE (P = 0.040), which meant that the mutant allele was not normally distributed in the studied population. In Saudi and non-Saudi subjects, the CYP3A5*3 allele was mutant homozygous (TT) in 13 and 3 participants with genotype frequencies of 6.80% and 3.53%, respectively, and wild homozygous (CC) in 114 and 70 participants with genotype frequencies of 60.40% and 82.35% and heterozygous (CT) and in 62 and 12 participants with genotype frequencies of 32.80% and 14.12%, respectively, with MAF of 0.233 and 0.106 respectively. In Saudi and non-Saudi subjects, the CYP3A4*22 allele was wild homozygous (GG) in 181 and 81 participants with genotype frequencies of 95.80% and 95.30%, respectively, and heterozygous (GA) in 8 and 4 participants with genotype frequencies of 4.20% and 4.70% with MAF of 0.021 and 0.024, respectively.

**Table 2:** Mutant allele and genotypes distribution of CYP3A4*22 (AA/GA) in total, Saudi and non-Saudi participants.

Genetic Polymorphism	Saudi			Non-Saudi			Total				
Genotypes	Number (n = 189)	Genotypes distribution (%)	MAF	Number (n = 85)	Genotypes frequencies	MAF	Number (n = 274)	Genotypes distribution (%)	MAF	χ2	HWE- *P* value
CYP3A4*22											
Mutant Homozygous (AA)	**-**	**-**	0.021	-	-	0.024	-	-	0.022	0.147	0.701
Wild Homozygous (GG)	181	95.80		81	95.30		262	95.60			
Heterozygous (GA)	8	4.20		4	4.70		12	4.40			
G	370	97.8		166	97.6	OR (95% CI) 0.89 (0.27–3.02)					
A	8	2.2		4	2.4						

For CYP3A4*22 rs35599367 (American MAF = 0.026, GG = 0.948, GA = 0.052/ South Asian MAF = 0.006, GG = 0.998, GA = 0.012/ European MAF = 0.050, GG = 0.903, GA = 0.095, AA = 0.002 /African MAF = 0.001, GG = 0.998, GA = 0.002).

Data were expressed as number (%). MAF: Minor allele frequency, χ2: Chi square, HWE. *P*—value: for the Hardy-Weinberg equilibrium.

**Table 3:** Mutant allele and genotypes distribution of CYP3A5*3 (TT/CT) in total, Saudi and non-Saudi participants.

Genetic Polymorphism	Saudi			Non-Saudi			Total				
Genotypes	Number (n = 189)	Genotypes distribution (%)	MAF	Number (n = 85)	Genotypes frequencies	MAF	Number (n = 274)	Genotypes distribution (%)	MAF	χ2	HWE- *P* value
CYP3A5*3											
Mutant Homozygous (TT)	13	6.80	0.233	3	3.53	0.106	16	5.80	0.193	4.23	0.040
Wild Homozygous (CC)	114	60.40		70	82.35		184	67.20			
Heterozygous (CT)	62	32.80		12	14.12		74	27.00			
C	290	77.00		152	89.00	OR (95% CI) 2.56 (1.49–4.41)					
T	88	23.00		18	11.00						

For CYP3A5*3 rs776746 (American MAF = 0.203, TT = 0.058, CC = 0.651, CT = 0.291/ South Asian MAF = 0.332, TT = 0.121, CC = 0.456, CT = 0.423/ East Asian MAF = 0.287, TT = 0.079, CC = 0.506, CT = 0.415).

Data were expressed as number (%). MAF: Minor allele frequency, χ2: Chi square, HWE. *P*–value: for the Hardy-Weinberg equilibrium.

### Frequencies of combined *CYP3A4/5* genotypes

Our results show that none of the participants were classified as PM because none of them were carriers of the mutant homozygous allele (AA), heterozygous (GA) for CYP3A4*22, or carriers of the mutant (TT) genotype allele for CYP3A5*3 (Table 4). However, 28 (10.00%) of the participants were classified as IMs and most of the participants 246 (90.00%) were classified as EMs. Among the Saudi participants, the frequencies of IMs and EMs were 21 (11%) and 168 (89%) respectively. Similarly, the Non-Saudi participants showed frequencies of 7 (8%) and 78 (92%) for IMs and EMs, respectively (Table 5).

**Table 4:** The effect of combined *CYP3A4/5* genotypes on metabolizers status among all participants.

Allelic status	*CYP3A4* DOF allele Carriers (AA) or (GA)	*CYP3A4* normal expresser allele (GG)
*CYP3A5* LOF allele (TT)	PMs	IMs
	N = 0 (0%)	N = 16 (5.7%)
*CYP3A5* expressers (CC) or (CT)	IMs	EMs
	N = 12 (4.3%)	N = 246 (90%)

N: number of participants, LOF: Loss of function, DOF: Decrease of function, PMs: Poor metabolizers, IMs: Intermediate metabolizers, EMs: Extensive metabolizers.

Data were expressed as numbers (%).

**Table 5:** The frequencies of intemediate and extensive metabolizers among Saudi and non-Saudi participants.

Metabolizers types	Total (n = 274)		Saudi (n = 189)		Non-Saudi (n = 85)	
	n	percentage	n	percentage	n	percentage
Poor metabolizers	-		-		-	
Intermediate metabolizers	28	10%	21	11%	7	8%
Extensive metabolizers	246	90%	168	89%	78	92%

### Effect of CYP3A4*22 and CYP3A5*3 genotypes on plasma simvastatin and lipid levels

The effect of combined *CYP3A4/5* genotypes on LDL-C and TC and simvastatin plasma levels in patients with hypercholesterolemia is shown in Table 6. A significant decrease (P <0.05) in simvastatin plasma levels was observed in EMs from 41.19 ± 7.20 to 65.16 ± 12.00 ng/ml in IMs. This resulted in a significant increase (P <0.05) in TC from 3.32 ± 0.70 in IMs to 5.12 ± 0.90 mmol/L in EMs. Similarly, LDL-C levels were significantly (P <0.05) increased from 1.90 ± 0.40 mmol/L in IMs to 3.25 ± 0.65 mmol/L in EMs.

**Table 6:** The Effect of combined *CYP3A4/5* genotypes on low density lipoprotein, total cholesterol and plasma simvastatin levels in hypercholesteremia patients.

Genotypes Combined *CYP3A4/5* genotypes	Number (%)	LDL-C reading mmol/L	TC reading mmol/L	Plasma statin concentration (ng/ml)
Intermediate metabolizers	15 (10%)	1.90 ± 0.40	3.32 ± 0.70	65.16 ± 12.00
Extensive metabolizers	133 (90%)	3.25 ± 0.65*	5.12 ± 0.90*	41.19 ± 7.20*

Data were expressed as mean +/- SEM or number (%) as appropriate.

## Discussion

In this study, the high frequencies of CYP3A4*22 (rs35599367) for the wild type homozygous (GG) in all studied groups, in Saudi participants and in non-Saudi participants were similar to those reported in the 1000 Genomes database. Our results showed allele frequencies for G = 0.978 and A = 0.022. Allele frequencies for other populations showed that African (G = 0.999, A = .001), American (G = 0.974, A = 0.026), European (G = 0.950, A = 0.050) and South Asians (G = 0.994, A = 0.006). Meanwhile, the homozygous mutant allele (AA) was not detected in either Saudi or non-Saudi participants which could be explained by its low allele frequency and its occurrence mainly in the Caucasian population [41, 42].

Global genotype frequencies of CYP3A5*3 (rs776746) reported in the 1000 Genomes database varied among different populations. The frequencies of wild homozygous (CC) ranged from 0.01 to 0.89, and mutant homozygous (TT) ranged from 0.02 to 0.78 among different populations [43]. Our results showed allele frequencies for C = 0.807 and T = 0.193. Allele frequencies for other populations showed that East Asians (C = 0.713, T = 0.287), American (C = 0.797, T = 0.203) and South Asians (C = 0.668, T = 0.332). The genotype frequencies of 0.67 for CC and 0.058 for TT for this SNP in all participants in our study were close to the genotype frequencies of East Asians, Americans and some South American populations. In addition, Lee and colleagues found that the MAFs for CYP3A5*3 (rs776746) were 0.255 in Korean, 0.085 in European-American, 0.198 in African-American, 0.344 in Han Chinese and 0.260 in Japanese [44]. It is observed that our study showed a MAF of 0.193 in all participants, which was close to that of African-American.

In our study, based on the combined *CYP3A4/5* genotype frequencies, it was considered that 90% of participants were EMs and only 10% were IMs. This is similar to previous studies that reported that most of the African population and African-Americans were classified as 90% EMs and 10% IMs [39, 42].

Depending on this study a higher dose should be prescribed for EMs patients, which supports the earlier study by Kitzmiller et al. which found that most PMs patients took lower doses of simvastatin, while, most EMs patients took higher doses of the statin [45].

We observed a significant difference in plasma statin concentrations in hypercholesterolemia patients between EMs and IMs in our study. In EMs the statin concentrations were 41.19 7.20 ng/ml which were significantly (P<0.05) decreased from 65.16 12.00 ng/ml in IMs. This resulted in a significant (P<0.05) increase in both LDL-C and TC. Other studies have also shown lower simvastatin concentrations in African-American carriers of the homozygous wild-type CYP3A5*1/*1 allele than in carriers of the mutant homozygous CYP3A5*3/3*, and similarly for the combined *CYP3A4/5* genotypes, plasma simvastatin concentration were found to be highest in PMs than IMs and least in EMs [39].

In addition, Elens et al. performed a study on patients on simvastatin, and found that patients who carried the CYP3A4*22 mutant homozygous allele (AA) had a better lipid lowering response to simvastatin than homozygous wild-type allele (GG) [46]. They also reported more reduction in TC and LDL-C levels in patients with DOF allele of CYP3A5*3 (TT or CT) than expresser allele (CC) carrier. Similar observations were shown by Kivisto et al. in Caucasian patients receiving simvastatin and reported that LDL-C and TC levels were higher in patients with CYP3A5*3 wild-type homozygous CC or heterozygous CT than in patients with mutant homozygous TT [47]. However, other studies of simvastatin users in Chinese patients found no association between rs35599367 in CYP3A*33 and rs776746 in CYP3A5*3 and phenotypes, and reported no significant differences between these SNPs and lipid-lowering response to simvastatin [48]. This was probably due to the low frequencies of these SNPs in the Chinese population. Similarly, studies on Greek hypercholesterolemia patients found no association between CYP3A4*22 allele and lipid-lowering response to simvastatin and atorvastatin and explained their findings due to the effects of some confounding or uncontrolled factors [49].

Our work has some limitations. The number of non-Saudi participants in the study was limited. Further work with more Saudi and non-Saudi participants (with similar Arab ethnicity) need to be conducted to support our findings. Further information on the functional implications of the different alleles in these SNPs is also lacking. Functional studies are important to clarify the exact role of these SNP’s in relation to drug response.

In conclusion, the current study shed light on the knowledge of two important SNPs in *CYP3A4/5* in Saudi and non-Saudi populations of Arab ethnicity, as these two CYPs are involved in the metabolism of various drugs including statins. A lower simvastatin dose should be administered to patients who have either a mutant homozygous allele for CYP3A5*3 or mutant homozygous and heterozygous alleles for CYP3A4*22. On the other hand, a higher dose of simvastatin should be administered to patients with either a homozygous wild-type allele for CYP3A5*3 or a homozygous wild-type allele for CYP3A4*22.

## Supporting information

S1 Table Demographic data and metabolism status. (PDF)

S2 Table Clinical data for statistical analysis. (PDF)

S1 Plot Alleles discrimination plot. (PDF)

## References

[1] Yusuf S, Hawken S, Ounpuu S, Dans T, Avezum A, Lanas F, et al. Effect of potentially modifiable risk factors associated with myocardial infarction in 52 countries (the INTERHEART study): case-control study. Lancet. 2004; 364: 937–952. doi: 10.1016/S0140-6736(04)17018-9 15364185

[2] Luque C, Cisternas FA, Araya M. [Changes in the patterns of disease after the epidemiological transition in health in Chile, 1950–2003]. Rev Med Chil. 2006; 134:703–712. doi: 10.4067/s0034-98872006000600005 17130944

[3] Benjamin EJ, Blaha MJ, Chiuve SE, et al. Heart Disease and Stroke Statistics-2017 Update: A Report From the American Heart Association [published correction appears in Circulation. 2017;135(10): e646] [published correction appears in Circulation. 2017;136(10): e196]. Circulation. 2017; 135(10): e146–e603. 10.1161/CIR.0000000000000485 PMC5408160 28122885

[4] Maniadakis N, Kourlaba G, Fragoulakis V. Self-reported prevalence of atherothrombosis in a general population sample of adults in Greece; a telephone survey. BMC Cardiovasc Disord. 2011; 11:16–25. doi: 10.1186/1471-2261-11-16 21492471 PMC3104943

[5] Waterworth DM, Ricketts SL, Song K, Chen L, Zhao JH, Ripatti S, et al. Genetic variants influencing circulating lipid levels and risk of coronary artery disease. Arterioscler Thromb Vasc Biol. 2010; 30: 2264–2276. doi: 10.1161/ATVBAHA.109.201020 20864672 PMC3891568

[6] Davila-Fajardo CL, Diaz-Villamarin X, Antunez-Rodriguez A, Fernandez-Gomez AE, Garcia-Navas P, Martinez-Gonzalez LJ, et al. Pharmacogenetics in the treatment of cardiovascular diseases and its current progress regarding implementation in the clinical routine. Genes 2019; 10, 26; doi: 10.3390/genes10040261 30939847 PMC6523655

[7] Musunuru K, Strong A, Frank-Kamenetsky M, Lee NE, Ahfeldt T, Sachs K, et al. From noncoding variant to phenotype via SORT1 at the 1p13 cholesterol locus. Nature. 2010; 466:714–719. doi: 10.1038/nature09266 20686566 PMC3062476

[8] Al Qahtani M, Al Backer T, Al Anazi T, Al Johani N, Binsalih S, Al Gobain M, et al. Impact of lipid disorders on mortality among Saudi patients with heart failure. J Saudi Heart Assoc. 2015; 27: 91–95. doi: 10.1016/j.jsha.2014.12.003 25870502 PMC4392377

[9] Barroso I. Genetics of Type 2 diabetes. Diabet Med. 2005; 22: 517–535. doi: 10.1111/j.1464-5491.2005.01550.x 15842505

[10] Levy D, Destefano AL, Larson MG, O’Donnell CJ, Lifton RP, Gavras H, et al. Evidence for a gene influencing blood pressure on chromosome 17. Genome scan linkage results for longitudinal blood pressure phenotypes in subjects from the framingham heart study. Hypertension. 2000; 36: 477–483. doi: 10.1161/01.hyp.36.4.477 11040222

[11] Ahmad A, Oparil S. Hypertension in women: recent advances and lingering questions. Hypertension. 2017; 70(1): 19–26. doi: 10.1161/HYPERTENSIONAHA.117.08317 28483918

[12] Samaan Z, Nowacki B, Schulze K, Magloire P, Anand SS. Smoking cessation intervention in a cardiovascular hospital based clinical setting. Cardiovasc Psychiatry Neurol. 2012;2012: 970108. doi: 10.1155/2012/970108 23097692 PMC3477663

[13] Tolfrey K. Intraindividual variability of children’s blood lipid and lipoprotein concentrations: a review. Prev Cardiol. 2002; 5:145–451. doi: 10.1111/j.1520-037x.2002.00563.x 12091757

[14] Mackay J, Mensah G. Atlas of Heart Disease and Stroke. Geneva, Switzerland: World Health Organization; 2004.

[15] Genest J, Frohlich J, Fodor G, McPherson R. Recommendations for the management of dyslipidemia and the prevention of cardiovascular disease: summary of the 2003 update. Cmaj. 2003; 169(9): 921–924. 14581310 PMC219626

[16] Theusch E, Chen YI, Rotter JI, Krauss RM, Medina MW. Genetic variants modulate gene expression in human lymphoblastoid cell lines. BMC Genomics. 2020; 21: 555. doi: 10.1186/s12864-020-06966-4 32787775 PMC7430882

[17] Vaughan CJ, Gotto AM Jr, Basson CT. The evolving role of statins in the management of atherosclerosis. J Am Coll Cardiol. 2000; 35: 1–10. doi: 10.1016/s0735-1097(99)00525-2 10636252

[18] Grundy SM. United States Cholesterol Guidelines 2001: expanded scope of intensive low-density lipoprotein-lowering therapy. Am J Cardiol, 2001; 88: 23J–27J. doi: 10.1016/s0002-9149(01)01931-2 11595195

[19] Ballantyne CM. Achieving greater reductions in cardiovascular risk: lessons from statin therapy on risk measures and risk reduction. Am Heart J. 2004;148(1 Suppl): S3–8. 15211326 10.1016/j.ahj.2004.04.025

[20] Davidson MH, Toth PP. Comparative effects of lipid-lowering therapies. Prog Cardiovasc Dis. 2004; 47: 73–104. doi: 10.1016/j.pcad.2004.04.007 15586350

[21] Davidson MH, Maki KC, Pearson TA, Pasternak RC, Deedwania PC, Mckenney JM, et al. Results of the National Cholesterol Education (NCEP) Program Evaluation ProjecT Utilizing Novel E-Technology (NEPTUNE) II survey and implications for treatment under the recent NCEP Writing Group recommendations. Am J Cardiol. 2005; 96: 556–563. doi: 10.1016/j.amjcard.2005.04.019 16098311

[22] Thom T, Haase N, Rosamond W, Howar VJ, Rumsfeld J, Manolio T, et al. Heart disease and stroke statistics—2006 update: a report from the American Heart Association Statistics Committee and Stroke Statistics Subcommittee. Circulation. 2006; 113: e85–151. doi: 10.1161/CIRCULATIONAHA.105.171600 16407573

[23] Roden DM, George AL Jr. The genetic basis of variability in drug responses. Nat Rev Drug Discov. 2002; 1: 37–44. doi: 10.1038/nrd705 12119608

[24] Schmitz G, Drobnik W. Pharmacogenomics and pharmacogenetics of cholesterol-lowering therapy. Clin Chem Lab Med. 2003; 41; 581–589. doi: 10.1515/CCLM.2003.088 12747606

[25] Ruiz-Iruela C, Padro-Miquel A, Pinto-Sala X, Baena-Diez N, Caixas-Pedragos A, Guell-Miro R, et al. KIF6 gene as a pharmacogenetic marker for lipid-lowering effect in statin treatment. PLoS one. 2018; 13 (10): e0205430. 10.1371/journal.pone.0205430 30304062 PMC6179259

[26] Guan Z, Wu K, Li R, Yin Y, Li X, Zhang S, et al. Pharmacogenetics of statin treatment: Efficacy and safety. J Clin Pharm Ther. 2019;44: 858–867. doi: 10.1111/jcpt.13025 31436349

[27] Novakova L, Vlckova H, Satinsky D, Sadilek P, Solichova D, Blaha M, et al. Ultra high performance liquid chromatography tandem mass spectrometric detection in clinical analysis of simvastatin and atorvastatin. J Chromatogr B Analyt Technol Biomed Life Sci. 2009; 877: 2093–2103. doi: 10.1016/j.jchromb.2009.05.052 19540175

[28] Zanger UM, Turpeinen M, Klein K, Schwab M. Functional pharmacogenetics/genomics of human *cytochromes P450* involved in drug biotransformation. Anal Bioanal Chem. 2008; 392: 1093–1108. doi: 10.1007/s00216-008-2291-6 18695978

[29] Kitzmiller JP, Sullivan DM, Phelps MA, Wang D, Sadee W. CYP3A4/5 combined genotype analysis for predicting statin dose requirement for optimal lipid control. Drug Metabol Drug Interact. 2013; 28: 59–63. doi: 10.1515/dmdi-2012-0031 23314529 PMC3681953

[30] Shitara Y, Sugiyama Y. Pharmacokinetic and pharmacodynamics alterations of 3-hydroxy-3-methylglutaryl coenzyme A (HMG-CoA) reductase inhibitors: drug-drug interactions and interindividual differences in transporter and metabolic enzyme functions. Pharmacol Ther. 2006; 112: 71–105. doi: 10.1016/j.pharmthera.2006.03.003 16714062

[31] Maggo SD, Kennedy MA, Clark DW. Clinical implications of pharmacogenetic variation on the effects of statins. Drug Saf. 2011; 34: 1–19. doi: 10.2165/11584380-000000000-00000 21142270

[32] Fiegenbaum M, Da Silveira FR, Van Der Sand CR, Van Der Sand LC, Ferreira M E, Pires RC, et al. The role of common variants of ABCB1, *CYP3A4*, and *CYP3A5* genes in lipid lowering efficacy and safety of simvastatin treatment. Clin Pharmacol Ther. 2005; 78: 551–558. doi: 10.1016/j.clpt.2005.08.003 16321621

[33] Becker ML, Visser LE, van Schaik RH, Hofman A, Uitterlinden AG, Ch. Stricker BH. Influence of genetic variation in CYP3A4 and ABCB1 on dose decrease or switching during simvastatin and atorvastatin therapy. Pharmacoepidemiol Drug Saf. 2010;19:75–81. doi: 10.1002/pds.1866 19802823

[34] Minstry of Health Annual Report 2018. Available from:. https://www.moh.gov.sa/en/Ministry/MediaCenter/Publications/Pages/Publications-2018-04-12-001.aspx

[35] Al-Nozha MM, Arafah MR, Al-Maatouq MA, Khalil MZ, Al-Marzouki K, Al-Mazrou YY, et al. Hyperlipidemia in Saudi Arabia. Saudi Med J. 2008; 29: 282–287. 18246242

[36] Medani KE, Al Mansour MA, Mohammed EY, Alfhaid F, Alghamdi TS, Sami W, et al. Prevalence and Risk Factors of Hypercholesterolemia in Majmaah, Saudi Arabia. Majmaah Journal of Health Sciences. 2018; Vol 6(1): 34–41.

[37] Williams JA, Ring BJ, Cantrell VE, Jones DR, Eckstein J, Ruterbories K, et al. Comparative metabolic capabilities of *CYP3A4*, *CYP3A5*, and *CYP3A7*. Drug Metab Dispos. 2002; 30: 883–891. doi: 10.1124/dmd.30.8.883 12124305

[38] Wang D, Sadee W. The Making of a CYP3A Biomarker Panel for Guiding Drug Therapy. J Pers Med. 2012; 2:175–191. doi: 10.3390/jpm2040175 24466438 PMC3901424

[39] Kitzmiller JP, Luzum JA, Baldassarre D, Krauss RM, Medina MW. *CYP3A4*22* and *CYP3A5*3* are associated with increased levels of plasma simvastatin concentrations in the cholesterol and pharmacogenetics study cohort. Pharmacogenet Genomics. 2014; 24: 486–491. doi: 10.1097/FPC.0000000000000079 25051018 PMC4160394

[40] Kurzawski M, Dabrowska J, Dziewanowski K, Domanski L, Peruzynska M, Drozdzik M. *CYP3A5* and *CYP3A4*, but not *ABCB1* polymorphisms affect tacrolimus dose-adjusted trough concentrations in kidney transplant recipients. Pharmacogenomics. 2014;15:179–188. doi: 10.2217/pgs.13.199 24444408

[41] Wang D, Guo Y, Wrighton SA, Cooke GE, Sadee W. Intronic polymorphism in *CYP3A4* affects hepatic expression and response to statin drugs. Pharmacogenomics J. 2011; 11: 274–286. doi: 10.1038/tpj.2010.28 20386561 PMC3248744

[42] Elens L, Van Gelder T, Hesselink DA, Haufroid V, Schaik RH. CYP3A4*22: promising newly identified *CYP3A4* variant allele for personalizing pharmacotherapy. Pharmacogenomics. 2013; 14: 47–62. doi: 10.2217/pgs.12.187 23252948

[43] The NHGRI-EBI catalog of human genome-wide association studies. Available from: https://www.ebi.ac.uk/gwas/home. 10.1093/nar/gkw1133 PMC5210590 27899670

[44] Lee JS, Cheong HS, Kim LH, Kim JO, Seo DW, Kim YH, et al. Screening of Genetic Polymorphisms of *CYP3A4* and *CYP3A5* Genes. Korean J Physiol Pharmacol. 2013;17: 479–484. doi: 10.4196/kjpp.2013.17.6.479 24381495 PMC3874433

[45] Kitzmiller JP, Binkley PF, Pandey SR, Suhy AM, Baldassarre D, Hartmann K. Statin pharmacogenomics: pursuing biomarkers for predicting clinical outcomes. Discov Med. 2013; 16: 45–51. 23911231 PMC4039562

[46] Elens L, Becker ML, Haufroid V, Hofman A, Visser LE, Uitterlinden AG, et al. Novel *CYP3A4* intron 6 single nucleotide polymorphism is associated with simvastatin-mediated cholesterol reduction in the Rotterdam Study. Pharmacogenet Genomics. 2011; 21: 861–866. doi: 10.1097/FPC.0b013e32834c6edb 21946898

[47] Kivisto KT, Niemi M, Schaeffeler E, Pitkala K, Tilvis R, Fromm MF, et al. Lipid-lowering response to statins is affected by *CYP3A5* polymorphism. Pharmacogenetics. 2004; 14: 523–525. doi: 10.1097/01.fpc.0000114762.78957.a5 15284534

[48] Hu M, Mak VW, Xiao Y, Tomlinson B. Associations between the genotypes and phenotype of *CYP3A* and the lipid response to simvastatin in Chinese patients with hypercholesterolemia. Pharmacogenomics. 2013; 14: 25–34. doi: 10.2217/pgs.12.181 23252946

[49] Ragia G, Kolovou V, Tavridou A, Elens L, Tselepis AD, Elisaf M, et al. No effect of *CYP3A4* intron 6 C>T polymorphism (*CYP3A4*22*) on lipid-lowering response to statins in Greek patients with primary hypercholesterolemia. Drug Metabol Personal Ther. 2015; 30: 43–48. doi: 10.1515/dmdi-2014-0021 25274942

