# Supplementary material for: Association of cytochromes P450 3A4*22 and 3A5*3 genotypes and polymorphism with response to simvastatin in hypercholesterolemia patients
Source: PLoS One. 2022 Jul 15;17(7):e0260824. doi: 10.1371/journal.pone.0260824 (PMC9286239; doi:10.1371/journal.pone.0260824)
Supplement: S2 Table — (PDF) [file pone.0260824.s002.pdf]

S2.Clinical Data

Column1	Age (year)	Sex	Nationality	Weight (Kg)	Hight (m)	Using Statins	Statin Dose (mg)	Sample #	Statin Plasma Conc. (ng/ml)	(mmol/L)	(mmol/L) (0 mmol/L)	rs35599367	rs776746	Sample #2
1	46	2	1	65.7	1.6	0		1				2	1	1
2	65	2	2	70.2	1.65	1	20	2	22.9	6.57	4.36	2	4	2
3	63	2	2	49	1.55	0		3				2	1	3
4	28	2	2	52	1.5	0		4				2	4	4
5	75	2	2	44.5	1.53	1	20	5	40.1	4.22	3.95	2	1	5
6	54	2	1	72	1.65	1	20	6	47.8	4.06	2.62	2	1	6
7	78	2	2	67	1.59	1	20	7	45.1	6.81	3.11	2	1	7
8	40	2	1	88	1.66	0		8				2	1	8
9	85	2	2	85	1.69	1	20	9	58.9	3.5	2.25	2	4	9
10	50	2	1	88.4	1.66	1	20	10	33.1	6.09	4.15	2	1	10
11	60	2	2	56.3	1.59	0		11				2	4	11
12	66	2	2	60.5	1.6	1	20	12	37.3	7.93	5.24	2	1	12
13	48	2	1	92	1.68	0		13				2	1	13
14	58	1	2	58	1.7	1	20	14	53.3	3.09	2.55	2	4	14
15	40	1	2	55.6	1.77	0		15				2	1	15
16	58	1	1	63	1.8	0		16				2	4	16
17	57	1	2	67	1.7	1	20	17	55.9	6.49	2.75	2	1	17
18	33	1	2	92	1.73	0		18				2	4	18
19	53	1	2	70	1.68	0		19				2	1	19
20	53	1	2	65	1.65	0		20				2	4	20
21	23	1	2	59	1.65	0		21				2	1	21
22	35	1	1	89	1.6	0		22				2	1	22
23	84	1	2	70	1.7	1	20	23	65.3	4.35	2.78	2	4	23
24	66	2	2	44	1.52	0		24				2	1	24
25	46	2	2	52.8	1.6	0		25				2	4	25
26	33	2	2	83.8	1.59	0		26				2	2	26
27	60	2	2	80	1.66	1	20	27	85.4	3.92	1.15	2	1	27
28	57	2	1	70	1.6	0		28				2	1	28
29	54	2	1	65	1.5	0		29				2	1	29
30	35	2	2	72.5	1.59	0		30				2	2	30

Column1	Age (year)	Sex	Nationality	Weight (Kg)	Hight (m)	Using Statins	Statin Dose (mg)	Sample #	Statin Plasma Conc. (ng/ml)	Total Cholesterol C (mmol/L)	LDL-C (mmol/L) (0 mmol/L)	rs35599367	rs776746	Sample #2
31	70	2	2	120	1.5	1	20	31	42.9	5.05	3.55	2	1	31
32	55	2	1	66.3	1.65	0		32				2	1	32
33	56	2	1	68.2	1.66	1	20	33	22.9	1.92	4.75	2	4	33
34	67	2	1	76.5	1.46	0		34				2	1	34
35	65	1	1	77	1.7	0		35				2	1	35
36	35	1	2	95.3	1.75	1	20	36	57.8	8.63	2.25	2	4	36
37	74	1	2	85.3	1.73	0		37				2	1	37
38	61	1	2	67	1.65	0		38				2	2	38
39	51	1	1	71	1.72	0		39				2	1	39
40	83	1	1	77.3	1.69	1	20	40	33.1	8.63	5.85	2	2	40
41	58	1	1	85	1.68	1	20	41	53.5	3.41	2.65	2	4	41
42	65	1	2	77.2	1.73	1	20	42	50.9	2.40	2.75	2	1	42
43	65	1	2	64.8	1.66	1	20	43	42.2	4.83	3.17	2	1	43
44	24	1	1	62.0	1.67	0		44				2	1	44
45	31	1	2	98.2	1.8	0		45				2	4	45
46	50	1	1	104	1.75	1	20	46	49.9	5.2	2.85	2	1	46
47	62	1	2	61	1.58	0		47				2	1	47
48	67	1	2	65.0	1.70	1	20	48	44.1	6.03	3.15	2	1	48
49	50	1	2	74	1.73	0		49				2	4	49
50	56	1	2	62.8	1.7	1	20	50	33.1	7.45	4.25	2	4	50
51	70	2	1	53.5	1.47	1	20	51	32.8	6.2	4.65	2	2	51
52	52	1	2	52.0	1.68	1	20	52	68.1	4.34	2.86	2	2	52
53	66	2	2	100	1.73	0		53				2	4	53
54	55	2	2	88.1	1.6	0		54				2	4	54
55	33	2	2	85.0	1.59	0		55				2	4	55
56	31	2	2	59.2	1.58	0		56				2	2	56
57	50	2	2	60	1.45	0		57				2	4	57

Column1	Age (year)	Sex	Nationality	Weight (Kg)	Hight (m)	Using Statins	Statin Dose (mg)	Sample #	Statin Plasma Conc. (ng/ml)	Total Cholesterol C (mmol/L)	LDL-C (mmol/L) (0 mmol/L)	rs35599367	rs776746	Sample #2
58	75	2	2	90	1.6	1	20	58	30.9	5.9	4.01	4	1	58
59	60	2	2	101	1.55	1	20	59	55.1	6.27	2.93	2	4	59
60	52	2	2	90	1.55	0		60				2	2	60
61	21	2	2	85	1.55	1	20	61	49.8	5.6	2.45	2	1	61
62	62	2	1	60	1.6	1	20	62	46.9	5.7	2.6	2	1	62
63	60	2	2	75.3	1.6	0		63				2	4	63
64	23	2	2	45	1.69	0		64				2	2	64
65	67	1	1	95	1.78	1	20	65	45.2	4.55	2.8	2	1	65
66	53	1	2	66	1.67	0		66				2	1	66
67	53	1	2	63.9	1.64	1	20	67	33.1	6.7	4.85	2	4	67
68	59	1	2	56.3	1.64	1	20	68	35.9	3.34	4.73	2	1	68
69	20	1	1	49	1.71	0		69				2	4	69
70	53	1	2	87	1.68	0		70				2	1	70
71	39	1	2	80	1.7	1	20	71	67.3	6.28	1.55	2	1	71
72	25	1	1	62	1.7	0		72				2	1	72
73	81	1	1	92	1.6	1	20	73	45.7	5.1	2.93	2	1	73
74	56	1	2	90	1.7	1	20	74	39.1	5.9	1.83	2	1	74
75	92	1	2	98.6	1.78	0		75				2	4	75
76	34	1	2	86	1.85	0		76				2	1	76
77	30	1	2	52.8	1.64	0		77				4	1	77
78	60	1	2	58.4	1.63	0		78				2	1	78
79	43	1	1	84.8	1.72	0		79				2	1	79
80	22	1	1	58	1.65	0		80				2	1	80
81	60	2	2	70	1.6	0		81				2	4	81
82	27	2	2	50	1.6	0		82				2	4	82
83	37	2	2	70	1.6	0		83				2	1	83
84	22	2	2	36	1.55	0		84				2	1	84

Column1	Age (year)	Sex	Nationality	Weight (Kg)	Hight (m)	Using Statins	Statin Dose (mg)	Sample #	Statin Plasma Conc. (ng/ml)	Total Cholesterol C (mmol/L)	LDL-C (mmol/L) (0 mmol/L)	rs35599367	rs776746	Sample #2
85	71	2	1	50	1.5	1	20	85	85.2	2.9	1.15	2	1	85
86	62	2	1	60.3	1.6	1	20	86	77.9	3.93	2.59	2	1	86
87	49	1	2	55.5	1.7	0		87				2	4	87
88	24	1	1	70.0	1.73	0		88				2	1	88
89	57	1	2	87.4	1.8	0		89				2	4	89
90	46	1	2	76	1.74	0		90				2	4	90
91	65	1	2	58	1.6	1	20	91	35.5	4.94	4.55	2	1	91
92	36	1	2	98.4	1.78	0		92				4	1	92
93	39	1	2	65	1.7	0		93				4	4	93
94	65	1	2	60	1.6	0		94				2	4	94
95	66	1	2	68.4	1.62	1	20	95	41.3	6.02	3.25	2	1	95
96	74	1	2	66.3	1.7	1	20	96	42.9	4.28	2.95	2	4	96
97	21	1	1	64.9	1.71	0		97				2	1	97
98	21	1	1	83	1.81	0		98				2	1	98
99	58	1	2	95	1.79	0		99				2	4	99
100	64	1	2	70	1.7	0		100				2	1	100
101	63	1	2	75	1.7	1	20	101	47.8	3.9	3.35	2	1	101
102	25	1	1	78	1.8	0		102				2	1	102
103	48	1	2	57	1.64	0		103				2	4	103
104	42	1	2	75.6	1.68	0		104				2	2	104
105	72	1	2	63	1.56	0		105				2	1	105
106	80	1	1	65	1.49	0		106				2	1	106
107	50	2	1	60	1.6	0		107				2	4	107
108	79	2	1	99	1.78	1	20	108	32.5	4.39	2.86	2	1	108
109	59	2	1	85	1.59	1	20	109	47.2	5.1	3.05	2	4	109
110	65	2	2	46.2	1.6	0		110				2	4	110
111	48	2	2	84	1.57	0		111				2	1	111
112	60	2	2	70	1.6	1	20	112	45.8	4.4	3.15	2	1	112
										Total Cholesterol C	LDL-C			

	Column1	Age (year)	Sex	Nationality	Weight (Kg)	Hight (m)	Using Statins	Statin Dose (mg)	Sample #	Statin Plasma Conc. (ng/ml)	Total Cholesterol C (mmol/L)	LDL-C (mmol/L) (0 mmol/L)	rs35599367	rs776746	Sample #2
119	113	34	2	2	62	1.5	0		113				2	1	113
120	114	54	2	2	50	1.48	0		114				2	4	114
121	115	40	2	2	100	1.66	1	20	115	49.1	5.63	3.84	2	4	115
122	116	69	2	2	88.5	1.59	1	20	116	35.7	3.48	3.75	2	1	116
123	117	65	2	2	77.4	1.58	1	20	117	38.4	5.08	2.84	2	4	117
124	118	42	2	2	100	1.65	1	20	118	62.8	7.3	1.49	2	4	118
125	119	60	2	1	69.4	1.63	0		119				4	1	119
126	120	25	2	1	57	1.53	0		120				2	1	120
127	121	25	2	1	82	1.57	0		121				2	1	121
128	122	25	2	1	86	1.68	0		122				2	4	122
129	123	36	2	2	101	1.63	0		123				2	1	123
130	124	31	2	1	54.1	1.58	0		124				2	1	124
131	125	30	2	1	80.5	1.61	0		125				2	1	125
132	126	20	2	1	53.5	1.59	0		126				4	1	126
133	127	24	2	1	61	1.62	0		127				2	1	127
134	128	37	1	2	108	1.83	0		128				2	1	128
135	129	23	1	2	81.9	1.83	0		129				2	1	129
136	130	24	2	1	50.2	1.56	0		130				2	1	130
137	131	19	2	1	56	1.69	0		131				2	1	131
138	132	65	2	2	70	1.57	0		132				2	1	132
139	133	31	2	2	81	1.49	0		133				2	1	133
140	134	27	2	1	73	1.45	0		134				2	1	134
141	135	34	1	1	80	1.67	0		135				2	1	135
142	136	28	1	2	78.5	1.68	0		136				2	1	136
143	137	31	1	2	79.3	1.7	0		137				2	1	137
144	138	34	1	1	89.7	1.76	0		138				2	1	138
145	139	43	1	1	91.6	1.76	0		139				2	4	139
146	140	27	2	1	64.5	1.56	0		140				2	1	140
											Total Cholesterol C	LDL-C			

	Column1	Age (year)	Sex	Nationality	Weight (Kg)	Hight (m)	Using Statins	Statin Dose (mg)	Sample #	Statin Plasma Conc. (ng/ml)	Total Cholesterol C (mmol/L)	LDL-C (mmol/L) (0 mmol/L)	rs35599367	rs776746	Sample #2
148	141	30	2	1	89.2	1.63	0		141				2	1	141
149	142	30	2	1	55.5	1.54	0		142				2	1	142
150	143	36	2	1	54.9	1.5	0		143				2	1	143
151	144	38	1	2	80.8	1.74	0		144				2	1	144
152	145	24	2	1	43.6	1.54	0		145				2	4	145
153	146	39	2	1	82.6	1.53	0		146				2	1	146
154	147	27	2	2	67.2	1.56	0		147				2	4	147
155	148	35	1	2	84.5	1.75	0		148				2	1	148
156	149	45	2	1	76.8	1.51	0		149				2	1	149
157	150	37	1	1	76.4	1.7	0		150				2	1	150
158	151	43	1	2	79.5	1.79	0		151				2	4	151
159	152	32	2	1	47	1.45	0		152				2	1	152
160	153	53	1	1	76	1.68	0		153				2	1	153
161	154	50	2	1	55.2	1.49	0		154				2	1	154
162	155	18	1	1	78	1.8	0		155				2	2	155
163	156	39	1	1	97	1.64	0		156				2	1	156
164	157	20	2	1	46	1.56	0		157				2	1	157
165	158	30	1	2	87.1	1.78	0		158				2	1	158
166	159	35	1	1	76.4	1.65	0		159				2	1	159
167	160	26	1	2	58.4	1.65	0		160				4	1	160
168	161	33	2	1	109.5	1.65	0		161				4	1	161
169	162	64	1	2	70	1.75	1	20	162	33.9	6.9	4.35	2	4	162
170	163	69	1	2	71	1.72	0		163				2	1	163
171	164	43	1	2	63	1.55	0		164				2	1	164
172	165	22	1	1	44.2	1.72	0		165				2	1	165
173	166	60	1	2	105	1.66	1	20	166	42.1	4.21	3.1	2	1	166
174	167	55	1	1	73	1.68	1	20	167	65.4	3.31	1.85	2	1	167
175	168	65	1	1	77.4	1.7	1	20	168	34.9	7.2	4.65	2	1	168

	Column1	Age	Sex	Nationality	Weight (	Hight	Using Statins	Statin Dose (	Sample #	Statin Plasma Conc. (ng	Total Cholesterol C (mm	LDL-C (mmol/L) (	rs35599367	rs776746	Sample #2
176	Column1	Age (year)	Sex	Nationality	Weight (Kg)	Hight (m)	Using Statins	Statin Dose (mg)	Sample #	Statin Plasma Conc. (ng/ml)	Total Cholesterol C (mmol/L)	LDL-C (mmol/L) (0 mmol/L)	rs35599367	rs776746	Sample #2
177	169	40	1	2	75	1.68	0		169				2	1	169
178	170	21	1	2	42	1.63	0		170				2	4	170
179	171	47	1	2	72	1.65	1	20	171	55.4	4.9	2.55	2	1	171
180	172	60	2	1	66	1.48	1	20	172	59.1	5.74	3.24	2	1	172
181	173	49	2	1	62	1.58	0		173				2	1	173
182	174	47	1	2	62	1.67	1	20	174	60.9	5.68	4.1	2	1	174
183	175	64	1	2	60.5	1.7	1	20	175	62.9	2.14	1.26	2	1	175
184	176	66	1	1	70.8	1.62	1	20	176	75.1	2.5	1.15	2	1	176
185	177	58	2	2	55.8	1.6	1	20	177	29.9	5.24	4.75	2	1	177
186	178	60	2	2	82	1.67	1	20	178	26.65	5.02	3.44	2	2	178
187	179	49	2	2	73.0	1.72	1	20	179	27.1	5.44	4.95	2	1	179
188	180	41	2	2	60	1.51	0		180				2	1	180
189	181	49	2	2	88.8	1.7	1	20	181	31.9	5.67	4.88	2	2	181
190	182	48	2	2	70.4	1.58	1	20	182	29.9	5.16	3.56	2	1	182
191	183	88	2	2	70	1.65	1	20	183	45.7	5.36	3.51	2	1	183
192	184	60	2	1	100	1.75	1	20	184	43.9	3.85	2.17	2	1	184
193	185	43	2	2	85	1.59	1	20	185	42.1	5.57	3.35	2	4	185
194	186	75	1	2	64	1.68	1	20	186	57.1	4.44	2.79	2	1	186
195	187	45	1	2	85	1.7	1	20	187	55.6	4.82	3.2	2	1	187
196	188	72	2	2	62	1.46	1	20	188	49.9	5.4	3.77	2	1	188
197	189	72	1	2	58.5	1.57	1	20	189	50.9	4.04	2.76	4	4	189
198	190	52	1	2	78	1.62	1	20	190	51.1	4.5	2.55	2	1	190
199	191	59	1	1	57	1.72	1	20	191	45.9	4.9	2.95	2	1	191
200	192	54	1	1	92.5	1.7	1	20	192	37.8	7.2	3.85	2	4	192
201	193	59	1	2	70.4	1.69	1	20	193	35.5	6.4	3.95	2	4	193
202	194	62	1	2	67.3	1.69	1	20	194	32.9	5.3	4.25	2	4	194
203	195	26	2	2	60.3	1.6	0		195				2	1	195
204	196	65	1	2	66	1.63	1	20	196	29.1	5.03	5.01	2	1	196

	Column1	Age	Sex	Nationality	Weight (	Hight	Using Statins	Statin Dose (	Sample #	Statin Plasma Conc. (ng	Total Cholesterol C (mm	LDL-C (mmol/L) (	rs35599367	rs776746	Sample #2
205	**Column1**	**Age (year)**	**Sex**	**Nationality**	**Weight (Kg)**	**Hight (m)**	**Using Statins**	**Statin Dose (mg)**	**Sample #**	**Statin Plasma Conc. (ng/ml)**	**Total Cholesterol C (mmol/L)**	**LDL-C (mmol/L) (0 mmol/L)**	**rs35599367**	**rs776746**	**Sample #2**
206	197	61	1	2	75	1.7	1	20	197	59.3	5.06	3.21	2	4	197
207	198	50	2	2	70.3	1.6	1	20	198	60.2	5.69	2.33	2	1	198
208	199	55	2	2	55	1.59	1	20	199	50.3	4.9	3.29	2	1	199
209	200	19	2	2	42	1.43	1	20	200	57.9	6.07	2.15	2	1	200
210	201	48	2	2	108	1.54	1	20	201	55.1	5.66	4.02	2	4	201
211	202	58	1	1	70	1.6	1	20	202	39.1	8.62	5.73	2	1	202
212	203	77	1	1	75	1.77	1	20	203	38.8	6.4	3.45	2	1	203
213	204	80	1	2	69.9	1.8	1	20	204	33.6	3.41	1.62	2	1	204
214	205	74	2	1	60	1.6	1	20	205	71.2	3.42	1.73	2	1	205
215	206	24	2	1	53.3	1.52	1	20	206	77.1	5.05	1.24	2	1	206
216	207	64	2	2	49	1.44	1	20	207	89.3	4.61	2.79	2	1	207
217	208	46	2	2	74	1.6	1	20	208	40.8	5.6	3.45	2	1	208
218	209	85	1	2	60.3	1.69	1	20	209	32.9	3.43	2.14	2	1	209
219	210	67	1	2	73.5	1.66	1	20	210	52.1	5.34	2.55	2	2	210
220	211	60	1	2	85	1.74	1	20	211	53.8	4.8	2.35	2	1	211
221	212	30	2	2	59.5	1.59	1	20	212	49.9	4.3	2.65	2	1	212
222	213	40	2	1	60.5	1.7	1	20	213	33.9	6.5	4.35	2	1	213
223	214	42	1	2	67	1,68	1	20	214	58.4	5.1	2.15	2	1	214
224	215	44	1	2	59.9	1.7	1	20	215	59.1	4.75	3.91	2	1	215
225	216	71	1	2	88	1.7	1	20	216	43.9	5.4	3.05	2	2	216
226	217	69	1	1	95	1.75	1	20	217	48.5	4.9	2.95	2	1	217
227	218	64	1	1	88.3	1.8	1	20	218	36.9	2.1	3.75	2	1	218
228	219	63	1	2	75.3	1.6	1	20	219	39.9	4.17	2.74	4	1	219
229	220	69	2	1	67	1.59	1	20	220	44.6	4.43	3.15	2	1	220
230	221	23	2	2	77.5	1.58	1	20	221	50.3	5.69	2.85	2	4	221
231	222	71	2	2	74.3	1.55	1	20	222	77.9	2.2	0.95	2	4	222
232	223	37	1	2	73.8	1.77	1	20	223	85.3	2.1	1.15	2	1	223
233	224	52	2	1	69.5	1.59	1	20	224	35.1	3.59	3.48	2	4	224
											[illegible]	[illegible]			

Column1 | Age | Sex | Nationality | Weight | Hight | Using Statins | Statin Dose (| Sample # | Statin Plasma Conc. (ng | Total Cholesterol C (mm | LDL-C (mmol/L) (| rs35599367 | rs776746 | Sample #2

234	Column1	Age (year)	Sex	Nationality	Weight (Kg)	Hight (m)	Using Statins	Statin Dose (mg)	Sample #	Statin Plasma Conc. (ng/ml)	Total Cholesterol C (mmol/L)	LDL-C (mmol/L) (0 mmol/L)	rs35599367	rs776746	Sample #2
235	225	75	2	2	60.4	1.59	1	20	225	29.7	6.7	4.95	2	1	225
236	226	54	2	1	55.3	1.66	1	20	226	22.9	6.35	3.68	2	4	226
237	227	50	1	2	80	1.7	1	20	227	51.3	4.7	2.25	2	1	227
238	228	53	2	2	117	1.5	1	20	228	45.9	5.52	3.05	2	1	228
239	229	70	2	2	52	1.6	1	20	229	35.5	4.85	3.55	2	1	229
240	230	70	2	1	58.4	1.64	1	20	230	32.9	8.51	5.75	4	1	230
241	231	50	1	2	82	1.7	1	20	231	44.8	4.82	3.15	4	1	231
242	232	89	1	2	60	1.72	1	20	232	72.9	2.9	1.25	2	1	232
243	233	62	1	2	111.2	1.6	1	20	233	85.9	2.75	1.15	2	4	233
244	234	75	1	2	70	1.7	1	20	234	82.8	7.12	1.39	2	4	234
245	235	60	2	2	90	1.59	1	20	235	58.6	5.83	2.35	2	1	235
246	236	72	1	2	100	1.75	1	20	236	75.9	5.11	1.61	2	1	236
247	237	63	2	2	81	1.58	1	20	237	60.7	6.43	2.85	2	1	237
248	238	60	2	2	85	1.6	1	20	238	70.9	3.33	1.86	2	1	238
249	239	51	2	2	66	1.62	1	20	239	65.2	4.67	2.48	2	1	239
250	240	60	1	1	70	1.75	1	20	249	24.31	7.3	4.85	2	1	240
251	241	65	1	1	91.5	1.75	1	20	250	44.86	4.67	3.05	2	1	241
252	242	65	1	2	70	1.6	1	20	251	41.11	4.93	3.1	2	4	242
253	243	60	2	2	60.3	1.65	1	20	252	24.82	5.9	4.65	2	4	243
254	244	62	2	2	88.4	1.68	1	20	253	18.37	7.8	4.95	2	4	244
255	245	63	2	2	87	1.62	1	20	254	65	5.53	1.95	2	1	245
256	246	57	2	2	60	1.65	1	20	255	23.69	6.12	4.18	2	4	246
257	247	63	1	2	100	1.7	1	20	256	88.2	2.6	0.95	2	4	247
258	248	55	1	2	124	1.68	1	20	257	33.55	6.7	3.95	2	1	248
259	249	50	2	2	72	1.64	1	20	258	65.1	4.17	3.07	2	1	249
260	250	50	1	2	61	1.68	1	20	259	70.2	3.32	1.85	2	1	250
261	251	55	2	2	82	1.7	1	20	260	98.3	2.23	1.52	2	4	251
262	252	63	1	2	81	1.8	1	20	261	102.68	2.1	0.75	2	1	252

	Column1	Age	Sex	Nationality	Weight (	Hight	Using Statins	Statin Dose (	Sample #	Statin Plasma Conc. (ng	Total Cholesterol C (mm	LDL-C (mmol/L) (	rs35599367	rs776746	Sample #2
263	**Column1**	**Age (year)**	**Sex**	**Nationality**	**Weight (Kg)**	**Hight (m)**	**Using Statins**	**Statin Dose (mg)**	**Sample #**	**Statin Plasma Conc. (ng/ml)**	**Total Cholesterol C (mmol/L)**	**LDL-C (mmol/L) (0 mmol/L)**	**rs35599367**	**rs776746**	**Sample #2**
264	253	57	2	2	58	1.48	1	20	262	33.6	7.99	5.39	2	4	253
265	254	60	1	2	62	1.55	1	20	263	41.9	5.41	2.22	2	1	254
266	255	58	1	2	51	1.64	1	20	264	55.8	3.35	2.1	2	4	255
267	256	81	1	2	62	1.62	1	20	265	33.9	6.3	4.25	2	4	256
268	257	73	2	2	80	1.59	1	20	266	29.1	5.29	2.1	2	1	257
269	258	60	2	2	80	1.59	1	20	267	45.45	4.5	2.56	2	4	258
270	259	50	2	2	82	1.5	1	20	268	22.34	8.51	5.6	2	1	259
271	260	64	1	2	64.3	1.6	1	20	269	55.2	4.24	2.55	2	1	260
272	261	70	1	2	74	1.65	1	20	270	20.32	5.46	4.15	2	1	261
273	262	50	1	2	110	1.77	1	20	271	16.65	8.5	4.25	2	4	262
274	263	68	1	2	82	1.65	1	20	272	19.75	7.9	4.64	2	1	263
275	264	67	1	2	72	1.5	1	20	273	19.92	8.3	4.5	2	1	264
276	265	69	1	2	70	1.63	1	20	274	77.4	2.43	1.67	2	1	265
277	266	33	1	2	95	1.7	1	20	275	29.9	6.5	4.55	2	1	266
278	267	74	1	1	75	1.7	1	20	276	39.1	4.9	2.65	2	1	267
279	268	55	1	2	75	1.6	1	20	277	29.9	6.9	4.76	2	4	268
280	269	53	1	2	75	1.75	2		278				2	1	269
281	270	54	1	2	89	1.65	2	20	279				2	1	270
282	271	71	2	2	65	1.65	1	20	280	33.9	6.5	2.74	2	1	271
283	272	47	1	2	89	1.7	1	20	281	29.9	8.1	3.43	2	1	281
284	273	39	1	2	69	1.64	1	20	293	44.7	5.59	3.26	2	4	293
285	274	61	2	2	50	1.55	1	20	294				4	4	294
286															
