# Supplementary material for: Association of cytochromes P450 3A4*22 and 3A5*3 genotypes and polymorphism with response to simvastatin in hypercholesterolemia patients
Source: PLoS One. 2022 Jul 15;17(7):e0260824. doi: 10.1371/journal.pone.0260824 (PMC9286239; doi:10.1371/journal.pone.0260824)
Supplement: S1 Plot — (PDF) [file pone.0260824.s003.pdf]

	A	B	C	D	E	F	G	H	I	J	K
1		**Plate1**					**reaction**				
2	**rs776746**										

S3. Alleles discrimination plot

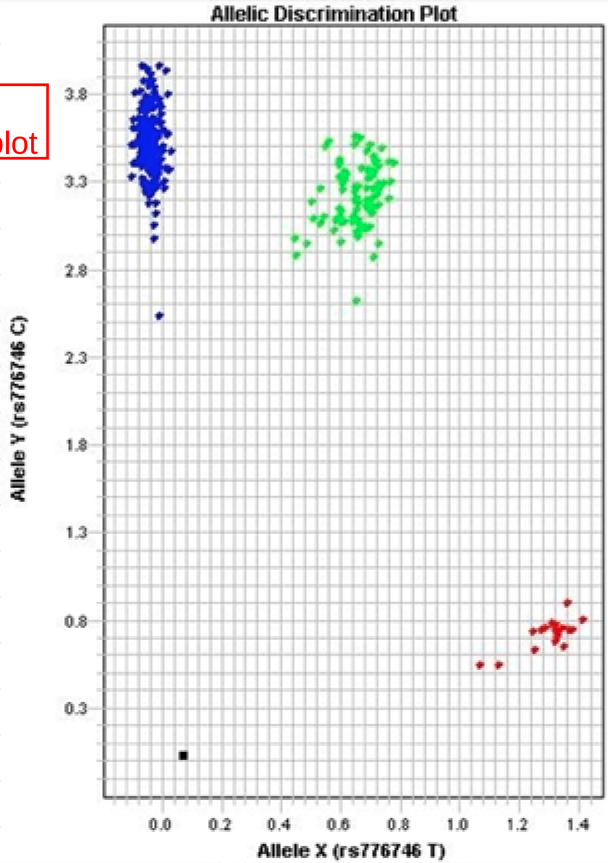

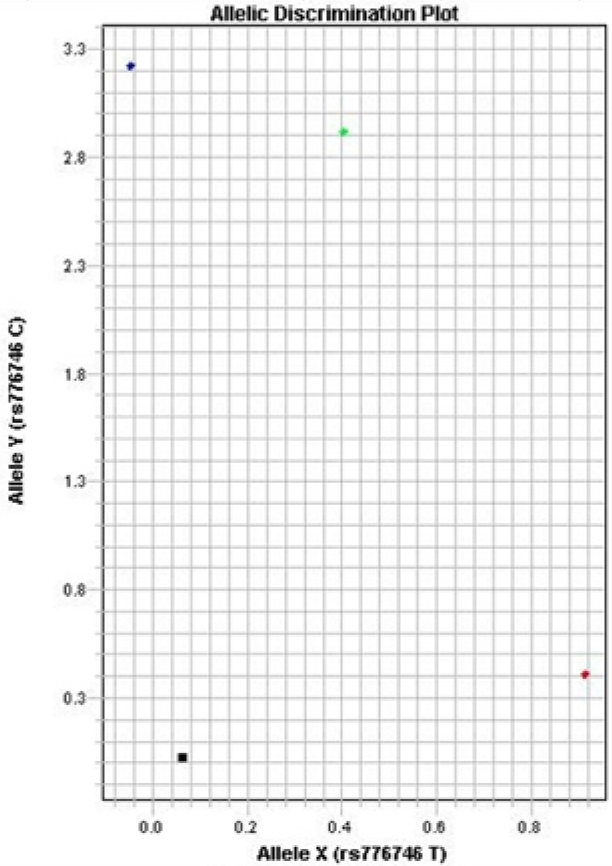

rs35599367

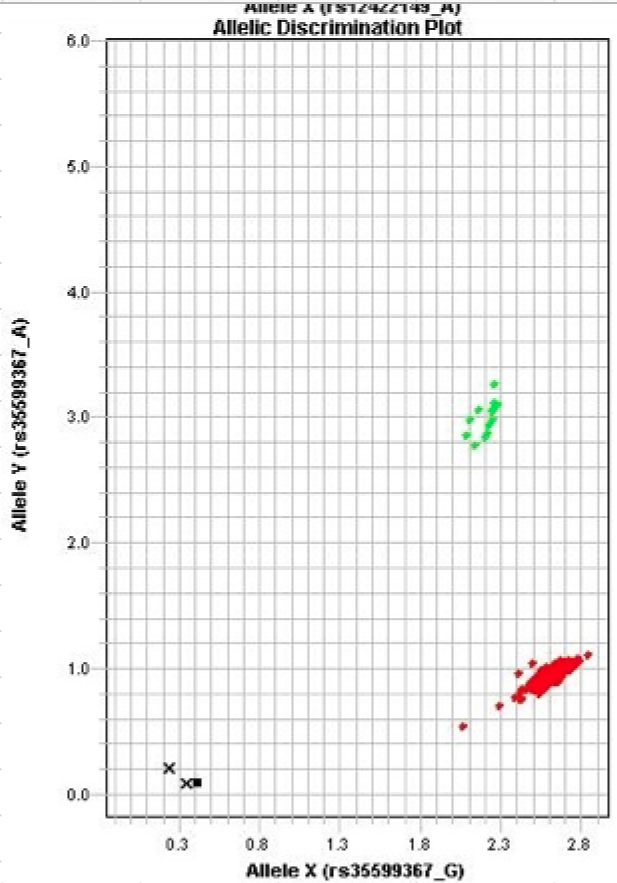
Allelic Discrimination Plot
6.0
5.0
4.0
3.0
2.0
1.0
0.0
Allele Y (rs35599367_A)
0.3
0.8
1.3
1.8
2.3
2.8
Allele X (rs35599367_G)

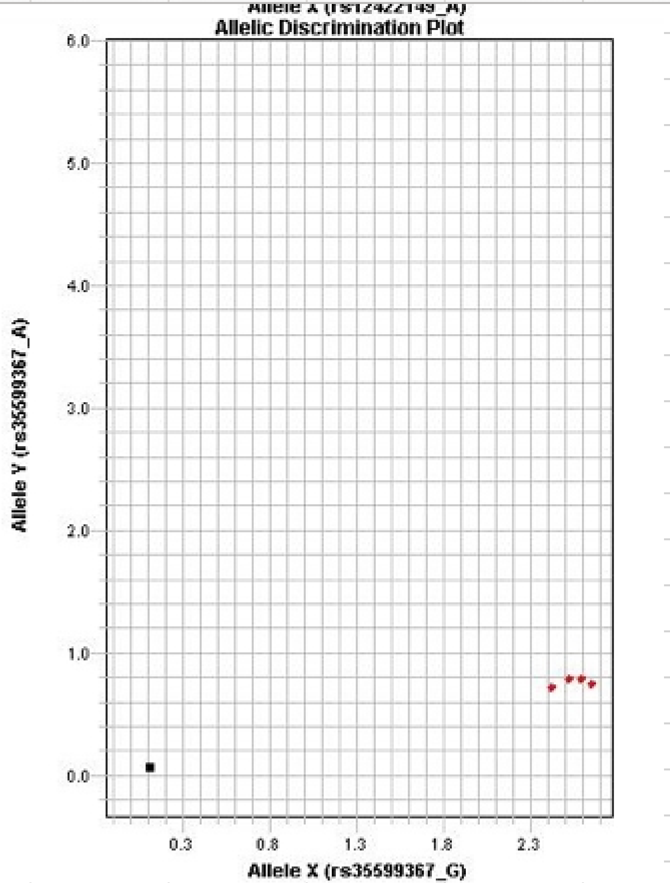
Allelic Discrimination Plot
6.0
5.0
4.0
3.0
2.0
1.0
0.0
Allele Y (rs35599367_A)
0.3
0.8
1.3
1.8
2.3
Allele X (rs35599367_G)
